# Applying the complex patient model to the care of persons with disabilities

**DOI:** 10.3325/cmj.2025.66.180

**Published:** 2025-04

**Authors:** Aleksandar Džakula, Karmen Lončarek, Tomislav Benjak, Darijo Jurišić, Dorja Vočanec

**Affiliations:** 1 Center for Health Systems, Policy and Diplomacy, University of Zagreb School of Medicine, Zagreb, Croatia *dvocanec@snz.hr*; 2 Center for Integrated and Palliative Care, University of Rijeka, Faculty of Medicine, Rijeka, Croatia; 3 Croatian Institute of Public Health, Zagreb, Croatia; 4 The Ombudsman for Persons with Disabilities, Zagreb, Croatia

The goal of every developed and modern society is to enable persons with disabilities to live as similarly as possible to other citizens. In most aspects of everyday life, including education and employment, persons with disabilities truly have the opportunity to develop and use their capacities and exercise their rights. However, a paradox often arises within the health care system when it comes to realizing these rights and promoting inclusion. Upon entering the health care system, persons with disabilities become patients. As a result, their status as patients is often prioritized, while their specific needs remain unmet.

In emergency and severe medical situations, the focus should be on treatment and recovery. However, persons with disabilities most frequently do not interact with the health care system in urgent or critical conditions when overlooking their specific needs could be justified.

## Unintentional neglect

The neglect of the specific needs of persons with disabilities cannot be attributed to intentional behavior by health care professionals or to any deliberate policy of the health care system. The reasons should be sought in a range of factors that shape the system’s environment and influence the behavior of health care workers in their everyday practice.

These two key determinants – the structure of the system and professional practice – must be thoroughly analyzed and placed at the center of all interventions aimed at improving the quality of health care. Understanding the specific needs of persons with disabilities carries strategic importance and must be continuously embedded into the very foundations of the system. Only in this way can we create an environment where persons with disabilities are not perceived solely as patients, but as individuals with their own characteristics, needs, and rights.

## A masked problem

The issue of appropriate care and access for persons with disabilities in the health care system is often masked by individual examples of good practice. Many persons with disabilities do receive high-quality care and proper treatment within specific segments of the health care system, most often in the parts of the system directly related to their primary condition or disability.

However, when persons with a disability require health care outside of that specialized area, they often enter a part of the system where their specific needs are neither recognized nor supported by adapted practices. As a result, the care they receive may fall short of being appropriate or inclusive.

## Errors in the basics

Persons with disabilities often struggle to effectively communicate their health concerns, either because health care professionals do not understand their specific needs or due to a lack of accessible communication channels. They also frequently encounter physical barriers within health care facilities or receive assistance in formats they cannot use (1,2). For example, individuals with severe visual impairments or difficulties understanding written text may be given instructions in small print, while those with hearing impairments may receive only verbal explanations without written follow-up. In some cases, patients are instructed to carry out parts of therapy or rehabilitation on their own, even though their physical, cognitive, or social limitations make this unfeasible. Children with autism spectrum disorder are often placed in overcrowded waiting rooms, where overstimulation may lead to a meltdown and prevent them from staying for the appointment.

These situations reduce the quality of the care provided, diminish the treatment effectiveness, and contribute to a sense of exclusion. The health care system must ensure flexible, individualized approaches that guarantee all patients equal access to effective and appropriate care.

## (In)effective interventions

Although policies and practices aimed at improving care for persons with disabilities are present throughout society, these efforts have not taken hold as strongly within the health care system. There are several possible explanations for this situation. One is that health care workers are inadequately educated about the needs of persons with disabilities (3,4). Another is that they rely on well-established routines and generalized practices applied uniformly to all patients, without sufficient adaptation to individual needs. Additionally, the health care system remains largely focused on medical aspects of care, lacking established practices for actively identifying and addressing broader and more specific patient needs. As a result, health care institutions and systems often lack clearly developed protocols and guidelines for managing patients with specific needs.

## Shifting the lens and approach – the complex patient model

In situations where the health care system, despite its capacity to care even for the most severe cases, fails to meet the specific needs of persons with disabilities, a key question arises: Can the health care system improve its approach to persons with disabilities?

The answer to this question lies in adopting a new perspective – the complex patient model. This approach, which acknowledges and identifies the multifaceted needs of patients, opens up new possibilities for improving the quality of care for persons with disabilities. By applying the complex patient model, health care institutions are given the opportunity to assess their capacity, capabilities, and preparedness to deliver adequate care to persons with disabilities even before they enter the system.

In the broader context of developing care for complex patients, programs targeting persons with disabilities are especially useful, as this group is already well-regulated. Comprehensive legislation supporting persons with disabilities has existed for years, along with a national registry that serves as a valuable tool for tracking, analysis, and policy planning (5).

Structuring and categorizing the needs of complex patients and developing targeted interventions represent a strategic opportunity for the health care system as a whole. Through this, tailored services for persons with disabilities can be actively managed and developed. For example, the introduction of a hospital passport for persons with autism spectrum disorder has shown that simple, low-cost, low-tech solutions can significantly enhance care for specific disability groups (6).

This approach would not only improve the overall quality of care for patients with disabilities but also ease the workload and reduce stress for health care professionals responsible for their care. Ultimately, the concept of complex patient care marks a professional, managerial, and humane step forward in redefining the relationship between the health care system and persons with disabilities.
